# Scientific Items

**Published:** 1882-10-20

**Authors:** 


### SCIENTIFIC ITEMS.

**Curious Fact Concerning Boiling Water.**—At a recent Association Meeting, Mr. A. J. Haddock, A. I. C., related the following: A kettle filled with boiling water was hung in the hottest room of some Turkish baths with the lid on. The temperature of the surrounding air was 252° Fahr. After about an hour the temperature of the water was taken, and indicated, as was expected, 242°. The kettle was then re-hung with the lid off. The temperature of the room was now 252°. In twenty minutes the temperature of the water had fallen to 185°, in thirty minutes to 178°, in forty-five minutes to 170°, and was evidently still falling. The manager stated that it generally fell finally to about 140°, when a point of equilibrium seemed to be established, and the water neither got hotter or cooler. Mr. Haddock supposes this loss of heat was due to rapid evaporation, and conversion of the sensible heat of the water into the latent heat of steam, and as dry air is a very bad conductor of heat (one of the worst known), the heat required to convert a portion of the water into steam had to be abstracted from the remainder of the water, thus lowering its temperature. In substantiation of this explanation, we know as a fact that if water is placed in a vessel over a large bulk of strong sulphuric acid, in the receiver of an air pump, and the air is exhausted, the rapid evaporation of one portion of the water will actually cause the rest to freeze.—*Mineral Water Trade Review.*

**Mica Masks.**—A well known German manufacturer of mica wares, Herr Raphael, of Breslau, now makes mica masks for the face, which are quite transparent, very light, and affected neither by heat nor by acids. They afford good protection to all workmen who are liable to be injured by heat, dust or noxious vapors, all workers with fire, metal and glass melters, stone masons, etc. In all kinds of grinding and polishing work the flying fragments rebound from the artificial mica plates of the mask without injuring them. These plates are fixed in a metallic frame, which is well isolated by means of asbestos, so as not to be attacked by heat or acid. These masks allow the turning of the eyes in any direction; and, as against mica spectacles, they afford the advantage of protection to the whole face. In certain cases the neck and shoulders may also be guarded by a sheet of cloth impregnated with fireproof material, or by an asbestos sheet attached to the mask. The interval between the mica and the eyes allows of workmen who have poor eyesight wearing spectacles, and of workers with fire or in melting operations wearing colored glass spectacles under the mask without fear of breakage of the glass, mica being such a bad conductor of heat. Where the mask has to be long worn, it is found desirable to add a caoutchouc tube, with a mouthpiece, for admission of fresh air; the tube passes out to the shoulders, where its funnel-shaped end (sometimes holding a moistened sponge) is supported. The mask has a sort of cap attached to it for fixture on the head.—*Druggists' Circular.*

**The Center of Population.**—The center of population in the United States was twenty-two miles from Baltimore in 1790, and has moved westward at the average rate of about fifty-one miles every decade, never deviating to the extent of a degree north or south of the thirty-ninth parallel. The greatest progress was between the years 1850 and 1860, when it traveled eigety-one miles from a point in Virginia to twenty miles south of Chillicothe, Ohio. This movement was caused by the settlement of the Pacific coast. The center of population in 1870 was forty-eight miles northwest of Cincinnati. According to the last census, the center had advanced westward fifty-eight miles, and deflected to the south about eight, being near the village of Taylorsville, Kentucky, about eight miles from Cincinnati. It is anticipated that the next census will find it in Jennings county, Indiana. Supposing the westward movement of population to continue, the central point should cross the Mississippi about 1890 not far from the mouth of the Missouri. It is considered probable, however, that it will never go so far westward, as there are large areas in the west which are only adapted for mining and grazing pursuits, and will support but a scanty population. The increase in the region beyond the Mississippi, after the close of the present century, may not much more than counterbalance that of the rest of the country, in which case the center of population will remain almost stationary in Southern Illinois.—*Mechanical News.*

**A Curious Bronze Coin** was recently picked up on his land by a Cass county, Illinois, farmer. It was sent to Prof. F. F. Hilder, of St. Louis, who makes the following report: "Upon examination I identified it as a coin of Antiochus IV., surnamed Epiphanes, one of the kings of Syria, of the family of the Seleucidæ, who reigned from 175 B. C. to 164 B. C., and who is mentioned in the Bible (first book of Maccabees, chapter 1, verse 10), as a cruel persecutor of the Jews. The coin bears on one side a very finely executed head of the king, and on the obverse a sitting figure of Jupiter, bearing in his extended right hand a small figure of Victory, and in his left a wand or scepter, with an inscription in ancient Greek characters—BASILEOS ANTIOCHOU, EPIPHANOUS, and another word, partly defaced, which I believe to be NIKEPHOROU; the translation of which is: King Antiochus, Epiphanes (Illustrious) the Victorious. When found it was very much blackened and corroded from long exposure, but when cleaned it appeared in a fine state of preservation and but little worn."—*Mechanical News.*

**Dynamogen** is the name of a new explosive, invented by M. Petri, a Vienna engineer. If the enthusiastic claims of the inventor are not an exaggeration of its merits, it will certainly prove a dangerous rival of gunpowder.—*Jour. of Chem.*

**Materialists** sometimes quote the words of Kant, "Give me matter, and I will explain the formation of a world;" but they omit his other words, "Give me matter only, and I cannot explain the formation of a caterpillar."—*Ibid.*

